# Molecular portrait-based correlation between primary canine mammary tumor and its lymph node metastasis: possible prognostic-predictive models and/or stronghold for specific treatments?

**DOI:** 10.1186/1746-6148-8-219

**Published:** 2012-11-12

**Authors:** Germana Beha, Barbara Brunetti, Pietro Asproni, Luisa Vera Muscatello, Francesca Millanta, Alessandro Poli, Giuseppe Sarli, Cinzia Benazzi

**Affiliations:** 1 Department of Veterinary Medical Sciences, University of Bologna, Via Tolaradi Sopra 50, Ozzano Emilia, Bologna 40064, Italy; 2 Department of Animal Pathology, School of Veterinary Medicine, Pisa, Italy

**Keywords:** Dogs, Mammary tumors, Molecular phenotypes, Lymph node metastasis, Concordance, Discordance

## Abstract

**Background:**

This study aimed to evaluate the relationship between the molecular phenotype of the primary mammary tumor and its related lymph node metastasis in the dog to develop prognostic-predictive models and targeted therapeutic options.

**Results:**

Twenty mammary tumor samples and their lymph node metastases were selected and stained by immunohistochemistry with anti-estrogen receptor (ER), -progesterone receptor (PR), -human epidermal growth factor receptor 2 (c-erbB-2), -cytokeratin 5/6 (CK 5/6), -cytokeratin 14 (CK14), -cytokeratin 19 (CK 19) and -protein 63 (p63) antibodies. Four phenotypes (luminal A, luminal B, c-erbB2 overexpressing and basal-like) were diagnosed in primary tumors and five (luminal A, luminal B, c-erbB-2 overexpressing, basal-like and normal-like) in the lymph node metastases. Phenotypic concordance was found in 13 of the 20 cases (65%), and seven cases (35%) showed discordance with different lymph node phenotypic profile from the primary tumor.

**Conclusions:**

The phenotype of the primary tumor assumes a predictive-therapeutic role only in concordant cases, meaning that both the primary tumor and its lymph node metastasis should be evaluated at the same time. A treatment plan based only on the primary tumor phenotype could lead to therapeutic failures if the phenotype of the lymph node metastasis differs from that of the primary tumor.

## Background

Canine mammary tumors and human breast cancer are heterogeneous diseases commonly occurring in female dogs [1,2] and in women [3,4].

Traditionally, breast cancer has been classified by morphological criteria in both human [5] and veterinary [6,7] medicine. Recent veterinary attention has focused on the protein expression profile [8,9] that seems to play a crucial role in human medicine in determining the clinical course, the use of chemoendocrine therapy [10], and treatment effects [4]. Sorlie et al. [11] examined the human expression profiles of 115 breast carcinomas identifying five subtypes: two hormone (oestrogen and/or progesterone) receptor-positive types (luminal-like A and luminal B) and hormone receptor-negative types [human epidermal growth factor receptor 2- overexpressing, basal-like, and unclassified (“normal-like”)]. Further studies have demonstrated the usefulness of immunohistochemistry in spite of biomolecular investigations to detect the five reported subtypes [12]. Based on classification of the human gene expression profile, four main carcinoma subtypes have been identified in canine species [8]: luminal A, luminal B, c-erbB-2 overexpressing and basal-like. Luminal-like phenotypes were characterized by ER and/or PR positivity and subgrouped into luminal A, negative for c-erbB-2, and luminal B, positive for c-erbB-2 respectively. Basal-like phenotypes were characterized by the absence of ER, PR and the expression of cytokeratin 5/6 and/or 14 [8,9,13]. C-erbB-2 positivity in the basal-like tumors characterizes the c-erbB-2 overexpressing group [14]. Sassi et al. [9] used a panel of antibodies to classify canine mammary carcinomas into three tumor subtypes (luminal-like A and B and basal-like) out of the five known in human medicine (no c-erbB-2 overexpressing or normal-like types were found in this study) and out of the four known in veterinary oncology (no normal-like was also detected in the Gama et al. [8] work).

Recently, molecular characterization in human breast cancer has also been applied to the metastasing lymph nodes [15]. The metastatic process is in fact the most urgent, important and difficult issue to approach in human [16] and animal cancer medicine. The relationship between the primary tumor and the lymph node metastasis from the same patient was studied by Wu et al. [15] to determine if satellite tumors are uniform or divergent in molecular properties and to provide new information of diagnostic and therapeutic significance [16].

Recent publications emphasized several similarities between human breast cancer and canine mammary tumor, such as the relative age at onset, incidence, risk factors, biological behaviour, metastatic pattern, histopathological and molecular features, metastases-associated expression profile [17], and response to therapy [18,19].

In human breast cancer therapies, endocrine therapy is added to chemotherapy in hormone receptor–positive subtypes; the c-erbB-2 overexpressing subtype is c-erbB-2 driven and appropriate for chemotherapy and c-erbB-2 targeted therapy such as trastuzumab. The basal-like subtypes of breast cancer, which are not responsive to endocrine therapy or trastuzumab, are entirely reliant on chemotherapy [20,21]. In canine mammary tumors, the therapeutic approach is mainly surgery, seldom with adjuvant chemotherapy, but no standard therapeutic protocol is available [22,23]. Receptor evaluation has been introduced to use an anti-estrogen therapy, whose side-effects include endometritis in bitches with ER negative tumors [24].

The aim of this study was to analyze the relationship between the primary mammary tumor and its lymph node metastasis based on immunohistochemical molecular characterization to develop the most specific prognostic-predictive models and targeted therapeutic options.

## Methods

### Samples

Specimens of mammary carcinomas from 20 female dogs were collected from the database of the Pathology Service of the Department of Veterinary Medical Science of Bologna University and from the Department of Animal Pathology of Pisa University.

The 20 dogs comprised 11 mixed breed, two German shepherd, one Yorkshire terrier, two English setter, two Doberman, one Maremma shepherd and one poodle. Dog ages ranged from six to 14 years with a mean age of 10.3 and a median of 10.5. Two-year follow-up survival data were available for 11 out of the 20 animals with invasive carcinomas included in the study. Overall survival time was defined as the time from the day of diagnosis until the day of death or last follow-up. All the latest data are summarized in Table 1.

**Table 1:** Individual data

**N°**	**AGE**	**BREED**	OS – 730days*
**1**	9	mixed breed	
**2**	12	German shepherd	
**3**	7	Yorkshire terrier	D (120)
**4**	6	English setter	D (540)
**5**	10	Dobermann	
**6**	13	mixed breed	
**7**	11	Maremma shepherd	A
**8**	13	mixed breed	
**9**	11	mixed breed	
**10**	11	mixed breed	A
**11**	8	Dobermann	D (600)
**12**	11	mixed breed	
**13**	8	mixed breed	D (320)
**14**	10	German shepherd	A
**15**	11	mixed breed	D (450)
**16**	14	mixed breed	A
**17**	10	mixed breed	A
**18**	10	English setter	D (60)
**19**	10	mixed breed	
**20**	11	poodle	

*OS= Over-survival time after 730 days; D= dead; A= alive.

Cases were selected based on both the primary mammary neoplasia and histological grade II (grade II: invasive carcinoma and metastases to regional lymph nodes) according to Gilbertson et al. [25]. No cases displayed systemic metastases. Samples were available as sections stained with hematoxylin and eosin and obtained from formalin-fixed and paraffin-embedded tissue block.

### Histological diagnosis and immunohistochemistry

Histological diagnosis was made according to the WHO classification system [6]. Seven consecutive 4μm-thick sections were cut from the paraffin blocks containing representative tumor samples and labeled by immunohistochemistry with the following antibodies: anti-ER, -PR, -c-erbB-2, -CK5/6, -CK14, -CK19, -p63. Data on the primary antibodies are summarized in Table 2.

**Table 2:** Primary antibodies, resources and dilutions used in immunohistochemistry

**ANTIBODY (−ANTI)**	**CLONE**	**MANUFACTURER**	**DILUTION**
ER	B-10	Abcam (Cambridge, UK)	1: 300
PR	PR4-12	Oncogene TM (Boston, MA, USA)	1: 100
c-erbB-2	Polyclonal	Dako (Glostrup, Denmark)	1: 250
Cytokeratins 5/6	D5/16B4	Zymed (South San Francisco, CA, USA)	1: 100
Cytokeratin 14	Ab-1 (LL002)	NeoMarkers (Fremont, CA, USA)	1: 300
Cytokeratin 19	BA17	Dako (Glostrup, Denmark)	1: 50
p63	4A4	Dako (Glostrup, Denmark)	1: 50

Sections were dewaxed in toluene and rehydrated. Endogenous peroxidase was blocked by immersion in H_2_O_2_ 0.3% in methanol for 20 min. Sections were then rinsed in Tris Buffer and antigen was retrieved with citrate buffer (2.1 g citric acid monohydrate/liter distilled water), pH 6.0 (except for CK 5/6 which use EDTA, pH 8.0), and heating for two 5-min periods in a microwave oven at 750 W, followed by cooling at room temperature for 20 min. All antibodies were incubated with the tissue sections overnight at 4°C, and their binding was revealed by a commercial streptavidin-biotin-peroxidase technique (LSAB Kit, Dako, Amsterdam, The Netherlands). Diaminobenzidine (0.05% for 10 min at room temperature) was used as chromogen. Slides were counterstained with Papanicolaou's hematoxylin.

As a negative control, the primary antibody was replaced with an irrelevant, isotype-matched antibody to control for non-specific binding of the secondary antibody. As positive controls to assess the cross-reactivity with canine tissues and the specificity of the immunohistochemical stain, sections of normal canine uterus (for anti-ER and -PR antibodies), canine skin (for anti-CK5/6, -CK14, -CK19 and -p63 antibodies) were used following the same protocols. A human poorly differentiated invasive ductal mammary carcinoma (kindly provided by P. Viacava, Department of Oncology, University of Pisa, Italy) known to react with c-erbB-2 antibody was used as positive control.

The staining result was classified semi-quantitatively with a dichotomous evaluation: positive or negative. The sample was considered positive:

• when presenting cytoplasmic stain in more than 1% of the invasive tumor cells for anti-CK-19, CK-5/6 and anti-CK14 antibodies [26];

• when presenting complete membranous stain in more than 10% of tumor cells for anti- c-erbB-2 antibody according to the Hercept-test [27];

• when presenting nuclear stain in more than 5% of tumor cells for anti-ER and anti-PR antibodies [28];

• when presenting nuclear stain in more than 10% of tumor cells for anti-p63 antibody [29].

### Molecular taxonomy

The application of the panel allowed cases to be grouped into five molecular subtypes according to an algorithm by Sassi et al. [9] modified as follows:

• Luminal-A: ER+ and/or PR+, c-erbB-2–, regardless of CK5/6, CK14, p63 staining.

• Luminal-B: ER+ and/or PR+, c-erbB-2+, regardless of CK5/6, CK14, p63 staining.

c-erbB-2 overexpressing: ER–, PR–, c-erbB-2+ regardless of CK5/6, CK14, p63 staining.

• Basal-like: ER–, PR–, c-erbB-2–, CK5/6+ and/or CK14+ and/or p63+.

• Normal-like: Negative to all markers.

## Results

### Diagnosis

Eight of the 20 primary tumors were classified as simple tubulopapillary carcinomas, eight as solid carcinomas, two as complex carcinomas and two as anaplastic carcinomas.

### Immunohistochemistry and molecular phenotypes

**Figure 1:**
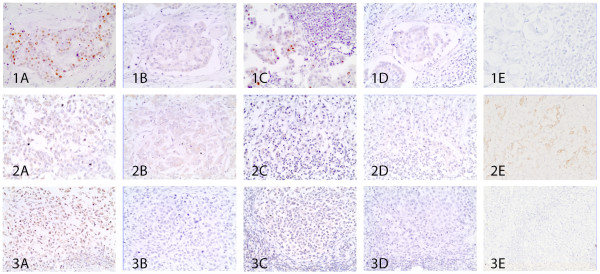
**Luminal A phenotype: concordant and discordant cases.** Line 1: Luminal A concordant case with ER+ and c-erbB-2– (1A, 1B) in the primary tumor and ER+, cerbB-2–, CK14– (1C, 1D, 1E) in the lymph node metastases. Line 2: Discordant case with luminal A phenotype PR+, c-erbB-2– (2A, 2B) in the mammary tumor and progression to basal-like phenotype PR–, c-erbB-2–, CK14+ (2C, 2D, 2E) in the respective nodal metastasis. Line 3: Discordant case presenting in the primary mammary carcinoma luminal A phenotype ER+, c-erbB-2– (3A, 3B) and normal-like phenotype ER–, cerbB-2–, CK5/6– (200x) (3C, 3D, 3E) in the lymph node. 400x.

**Figure 2:**
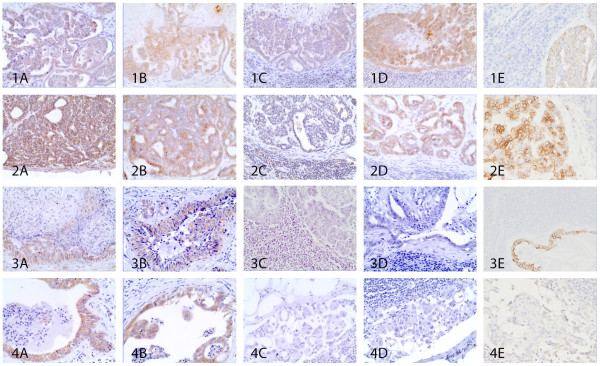
**Luminal B phenotype: concordant and discordant cases.** Line 1: Luminal B concordant case with PR+ and c-erbB-2+ (1A, 1B) in theprimary neoplasia and PR+, cerbB-2+, CK14+ (1C, 1D, 1E) in the lymph node metastases. Line 2: Discordant case showing luminal B phenotype PR+, c-erbB-2+ (2A, 2B) in the primary tumor becoming c-erbB-2 overexpressing phenotype in the lymph node PR–, c-erbB-2+, CK5/6+ (2C, 2D, 2E). Line 3: Discordant case with luminal B phenotype ER+, c-erbB-2+ (3A, 3B) in the mammary tumor and progression to basal-like phenotype ER–, c-erbB-2–, CK 14+ (3D, 3E, 3F) in the respective nodal metastasis. Line 4: Discordant case presenting in the primary mammary carcinoma luminal A phenotype ER+, c-erbB-2– (4A, 4B) and normal-like phenotype PR–, cerbB-2–, p63– (4D, 4E, 4F) in the lymph node. 400x.

**Figure 3:**
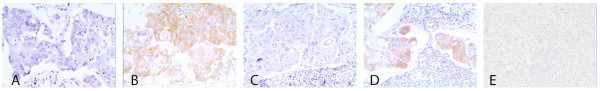
**C-erbB-2 overexpressing phenotype: concordant case.** PR–, c-erbB-2+ (1A, 1B) in mammary tumor and PR–, c-erbB-2+, p63– (200x) (2C, 2D, 2E) in the respective lymph node metastasis. 400x.

**Figure 4:**
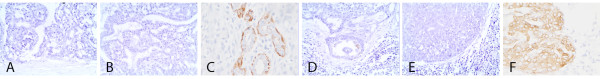
**Basal-like phenotype: concordant case.** ER–, c-erbB-2–, CK5/6+ (1A, 1B, 1C) in mammary neoplasia and PR–, c-erbB-2–, CK 14+ (2C, 2D, 2E) in the lymph node metastasis. 400x.

Immunohistochemistry for ER, PR, c-erbB-2, CK5/6, CK14, p63 in the primary tumor and in the respective lymph node metastasis is summarized in Table 3. In each case the epithelial origin of cancer was confirmed by CK19 staining. Based on the applied algorithm, molecular phenotypes were obtained in the primary mammary tumors and in their lymph node metastases (Table 4). Four phenotypes (luminal A (Figure 1A-B, line 1-2-3), luminal B (Figure 2A-B, line 1-2-3-4), c-erbB-2 overexpressing (Figure 3A-B), basal-like (Figure 4A-B-C)) were diagnosed in primary tumors (eight (40%), seven (35%), two (10%), three (15%) respectively) and five (luminal A (Figure 1C-D-E, line 1), luminal B (Figure 2C-D-E, line 1), c-erbB-2 overexpressing (Figure 2C-D-E, line 2 and Figure 3C-D-E), basal-like (Figure 2C-D-E, line 3 and Figure 4C-D-E), normal-like (Figure 1C-D-E, line 3 and line 4 of Figure 2C-D-E)) in the lymph node metastases (five (25%), three (15%), four (20%), six (30%), two (10%) respectively).

**Table 3:** Summary of immunohistochemical staining

**SAMPLES ID**	**PRIMARY MAMMARY TUMOR**						**LYMPH NODE METASTASIS**					
	ER*	PR*	**c-erbB-2°**	CK 14*	CK 5/6*	p63*	ER*	PR*	**c-erbB-2°**	CK 14*	CK 5/6*	p63*
**1**	1	1	1	1	1	0	0	1	1	1	1	0
**2**	0	1	1	1	1	0	0	1	1	1	1	0
**3**	1	0	1	1	1	1	0	0	0	0	0	0
**4**	0	0	1	1	1	0	0	0	1	1	1	0
**5**	0	1	3	1	1	1	0	1	3	1	1	0
**6**	1	0	1	1	1	1	0	0	1	1	1	1
**7**	1	1	1	1	1	1	1	1	0	1	1	0
**8**	0	1	1	1	1	0	0	0	1	1	1	0
**9**	1	1	3	1	1	1	0	0	3	1	1	0
**10**	1	1	3	1	1	1	1	1	3	1	1	0
**11**	0	0	1	1	0	0	0	0	1	1	0	0
**12**	1	0	2	1	1	1	0	0	1	1	1	0
**13**	1	0	2	1	1	1	0	0	1	0	0	0
**14**	1	1	1	1	1	0	1	0	1	1	1	0
**15**	0	0	2	1	1	0	0	0	3	1	1	0
**16**	1	0	1	1	1	0	1	0	1	0	1	0
**17**	0	1	2	1	1	0	0	1	2	1	1	0
**18**	0	0	2	1	1	1	0	0	2	0	0	0
**19**	0	0	1	1	1	0	0	0	1	1	1	0
**20**	0	1	2	1	1	1	0	0	2	1	1	1

*ER, PR, CK5/6, CK14, p63: 0= no immunoreactivity; 1= immunoreactivity. °c-erbB-2: 0= no immunoreactivity; 1= <10% positive cells; 2= 10 to 90% complete membranous immunostaining; 3= >90% complete membranous cell positivity.

**Table 4:** Molecular phenotypes

**SAMPLES ID**	**PHENOTYPE**		**PHENOTYPIC RELATIONSHIP**
	**PRIMARY TUMOR**	**LYMPH NODE METASTASIS**	
	**PRIMARY TUMOR**	**LYMPH NODE METASTASIS**	
**1**	Luminal A	Luminal A	Concordance
**2**	Luminal A	Luminal A	Concordance
**3**	Luminal A	Normal-like	Discordance
**4**	Basal-like	Basal-like	Concordance
**5**	Luminal B	Luminal B	Concordance
**6**	Luminal A	Basal-like	Discordance
**7**	Luminal A	Luminal A	Concordance
**8**	Luminal A	Basal-like	Discordance
**9**	Luminal B	c-erbB-2 overexpressing	Discordance
**10**	Luminal B	Luminal B	Concordance
**11**	Basal-like	Basal-like	Concordance
**12**	Luminal B	Basal-like	Discordance
**13**	Luminal B	Normal-like	Discordance
**14**	Luminal A	Luminal A	Concordance
**15**	c-erbB-2 overexpressing	c-erbB-2 overexpressing	Concordance
**16**	Luminal A	Luminal A	Concordance
**17**	Luminal B	Luminal B	Concordance
**18**	c-erbB-2 overexpressing	c-erbB-2 overexpressing	Concordance
**19**	Basal-like	Basal-like	Concordance
**20**	Luminal B	c-erbB-2 overexpressing	Discordance

### Relationship between molecular phenotype in the primary mammary tumor and its related lymph node metastasis

Phenotypic concordance was found in 13 of the 20 cases (65%) (five luminal A (Figure 1, line 1), three luminal B (Figure 2, line 1), two c-erbB-2 overexpressing (Figure 3) and three basal-like (Figure 4)). Seven cases (35%) showed discordance with lymph node phenotypic profile differing from the primary tumor (two luminal A became basal-like (Figure 1, line 2), one luminal A became normal-like (Figure 1, line 3), two luminal B became c-erbB-2 overexpressing (Figure 2, line 2), one luminal B became basal-like (Figure 2, line 3), one luminal B became normal-like (Figure 2, line 4)) (Table 3). C-erbB-2 overexpressing and basal-like primary tumors were 100% concordant with the molecular phenotype of the respective lymph node metastases (Figure 3 and Figure 4). Luminal A and luminal B were concordant in 65.2% and 42.9% respectively of primary tumors for the same comparison.

### Histological diagnosis in primary mammary tumor compared to molecular phenotypes and to concordance/discordance

In the primary tumor the luminal A phenotype displayed a different histological tumor pattern, i.e. simple tubulopapillary (one case) (Figure 1A-B, line 1), solid (six cases) (Figure 1A-B, line 2), anaplastic (one case) (Figure 1A-B, line 3). Luminal B phenotype displayed a different histological tumor pattern, i.e. simple tubulopapillary (four cases) (Figure 2A-B, line 1 and 4), solid (two cases) (Figure 2A-B, line 2) and complex (one case) (Figure 2A-B, line 3). The c-erbB-2 overexpressing phenotype showed two patterns, i.e. tubulopapillary (one case), and anaplastic (one case) (Figure 3A-B). The basal-like phenotype displayed different histological tumor patterns, i.e. simple tubulopapillary (two cases) (Figure 4A-B-C) and complex (one case). No association was found between histological diagnosis and phenotype in the primary tumor (Pearson Chi-square, P=0.065; for statistics only the tubulopapillary and the solid carcinomas were considered because the other two types had only one case for phenotype).

Six cases with tubulopapillary pattern displayed concordance between the primary tumor and its related lymph node metastasis whereas the other two cases showed discordance. In the solid pattern concordance was found in five cases and discordance in three. In the anaplastic and complex patterns both concordance and discordance were present with one case for each type. Comparing the four histological patterns, no differences in the percentages of concordance/discordance were evident (Pearson Chi square P=0.857, for statistics only the tubulopapillary and the solid carcinomas were considered because the other two types had only one case for each phenotype).

### Influence of the molecular phenotype and concordance/discordance on dog’s survival rate

Table 1 reports the survival data of the 11 animals. Few data are available to group the subjects according to molecular phenotypes and concordance/discordance to perform survival analysis even though it appears that all five animals alive at 24 months post-surgery were concordant luminal A or luminal B cases. The other six dead animals bore primary tumor/lymph node concordant (2 basal-like and 2 c-erbB-2 overexpressing) or discordant (1 luminal A/normal like; 1 luminal B/normal-like) cases in which less differentiated molecular types were present in both sites or only in the lymph node compared to the live animals.

## Discussion

Canine mammary carcinomas can become fatal due to the development of distant metastases. One of the most important prognostic factors in the diagnosis is the acknowledgment of metastases to the regional lymph node that represents an early step in metastatic spread [30]. Klopfleisch et al. [17] and Lu et al. [31] demonstrated that metastatic spread of canine mammary tumor to the lymph nodes is associated with a gene expression profile of increased cell cycle progression, altered cell differentiation and decreased growth factor signaling. Metastasis development is a complex process involving invasion, intravasation, survival in the bloodstream, extravasation and homing and proliferation at the site of metastasis [32]. Although some phenotypes showed greater aggressiveness and metastatic capability, only a selected subpopulation was able to metastatize in the multiple and heterogeneous tumor cell population. In this case the phenotype may have been transient and these selected cells have had an intrinsic program to transition to a phenotype enhancing their ability for heterotypic interaction and survival proliferation in distant organs [32] as Darwinian clonal evolution. Conversely, the metastatic process has also been described as a stochastic event, the primary tumor cells having equal metastatic capability, characterized by a phenotypic overlap between the primary tumor and its metastases [33,34]. Thus the identification of molecular phenotypes in primary tumors and metastases can provide predictive information on the most likely metastatic profile, not the condition in the primary tumor.

Sassi et al. [9] identified three phenotypes out of the four detected by Gama et al. [8], demonstrating that basal-like subtypes were associated with a better outcome than luminal A and luminal B tumors, in contrast with the findings of Gama et al. [8]. The prognostic role of c-erbB-2 overexpression remains controversial despite what is known in human medicine. According to a study by Hsu et al. [35], the relationship between the clinical course and protein expression of c-erbB-2 in dogs with malignant mammary neoplasia indicated a greater survival rate in tumors overexpressing c-erbB-2 compared to those having non-overexpressed levels of antigen. Certainly, c-erbB-2 plays an important role in carcinogenesis, but does not seem to be directly correlated with progression to malignancy [35]. In the present investigation it seems that luminal A or B concordance should be considered a positive prognostic factor, whereas concordance for the other molecular types or discordance should not, even if these results await confirmation in a larger number of cases and proper statistical analysis.

This study revealed four out of the five protein expression phenotypes of breast cancer in primary tumors (20 cases): luminal A (40%), luminal B (35%), c-erbB-2 overexpressing (10%) and basal-like (15%). The prevalence of luminal phenotypes (75%) over the others (25%) is in accordance with findings both in human [11,36,37] and veterinary [8,9] medicine.

Based on the present study and in agreement with Brunetti et al. [38], labeling for CK and p63 would only appear necessary when a tumor is negative for ER, PR and c-erbB-2.

With regard to luminal A and B phenotypes, the expression profiles of ER and PR are essential to decide on the application of endocrine therapy [39] in breast cancer and canine mammary neoplasia, and also seem to play a minor role in predicting tumor biological behaviour [40,41]. Wu et al.’s study [15] in breast cancer confirmed the observation that ER and/or PR could be lost when carcinomas metastasizes, thereby resisting endocrine therapy. The present study shows almost overlapping results, losing hormone receptors by moving from luminal A to basal-like (2 cases) and/or to normal-like (1 case) phenotypes and from luminal B to c-erbB-2 overexpressing (2 case) and/or to basal-like (1 case), and/or to normal-like (1 case), confirming that the gene expression profile in canine mammary tumors may prove a helpful tool in clinical practice. Chang et al. [42] indicated that the ER or PR expression in dogs was associated with tumor size, clinical stage, and lymph node metastasis or distant metastases. Dogs with malignant mammary neoplasia and expression of both ER and PR had a longer survival rate than dogs with malignant mammary tumors that were ER positive but PR negative. This latest information on PR suggested that the receptor was a better outcome predictor than ER status alone and that its positive or negative expression could serve as a prognostic factor, especially in dogs with malignant neoplasia with ER expressed [42]. The present results disclosed a high prevalence of hormone receptor expression in the primary tumor, whose positivity was ensured by reactivity to least one of the two markers (ER and/or PR) (Table 3). The latest results indicate that there are grounds for the use of anti-hormone therapy in dogs, administering molecules other than those hitherto used in veterinary medicine (tamoxifen) as their side-effects are already well-known [24]. A similar analysis in lymph node showed a net loss of hormone receptor expression, namely ER. ER loss is a known adverse prognostic factor [42], and therefore its lack of expression in metastases is indicative. In this study, only five out of the 20 cases showed positivity to both ER and PR in the primary tumor, with persistent positivity in the lymph node metastasis in only two cases. The remaining three cases showed loss of one or both hormone receptor staining in the lymph node metastasis: loss of ER (case n° 1), loss of PR (case n° 14) and concomitant loss of ER and PR (case n° 9). According to the literature, these cases should have a poor prognosis, justified by our phenotypes, but with maintained luminality in the first two cases, and a shift to c-erbB-2 overexpression in the third meaning a even worse evolution by the complete loss of ER and PR.

Interestingly, cases n° 3 and n° 13 (Table 3) in which in lymph node metastasis occurred, lost all the markers expressed in the primary tumor, likely due to the selection of a significantly aggressive cell subpopulation. The normal-like or multiple markers negative (MMN) subtype tumors have been shown to be negative for basal markers, such as CK5/6 and CK14 in our cases, and negative for other molecular markers. The majority of normal-like subtype tumors express CK8/18, CK19 (used in this study), with an absence of CK5/6 suggesting that these cells were most probably derived from a luminal gland cell [43]. The normal-like subtype is also included in the triple negative breast cancer group (TNBC) characterized by an aggressive clinical course, poor survival rate and, unlike the overexpressing hormone receptors or c-erbB-2-overexpressing tumors, is not amenable to hormone therapy or c-erbB-2-directed agents [44]. Although no correlation has been found between histological type and phenotype, the normal-like cases represent an exception.

The present study identified five phenotypes in the lymph node metastases: luminal A (25%), luminal B (15%), c-erbB-2 overexpressing (20%), basal-like (30%) and normal-like (10%). The novel aspect of this study is the evaluation of the lymph node metastasis phenotypes and their correlation with the primary tumor, never hitherto applied to canine species. The relationship between the primary tumor and metastatic phenotype is defined by a concordance in 65% of cases and a discordance in the remaining 35%, suggesting the two main metastatic capability theories coexist. All seven discordant cases showed a progressive behavior, according to the prognostic value of molecular phenotypes reported by Gama et al. [8], suggesting phenotypic evolution with a worse prognosis from the primary tumor to lymph node metastasis.

## Conclusions

Molecular phenotype classification is a new model urgently needed in veterinary medicine. This model will fill current gaps regarding prognosis and a targeted therapeutic approach, since the primary tumor phenotype does not always overlap with that of its metastasis. According to the present findings, the primary tumor phenotype assumes a predictive-therapeutic role only in concordant cases, meaning that there should be a concomitant evaluation of both the primary tumor and its lymph node metastasis. Treatment planning based only on the primary tumor phenotype can lead to therapeutic failures if the lymph node metastatic phenotype differs from that of the primary tumor.

## Competing interests

The authors declare that they have no competing interests.

## Authors’ contributions

These authors contributed equally to this work. All authors read and approved the final manuscript.
